# Clinical validation of a contactless respiration rate monitor

**DOI:** 10.1038/s41598-023-30171-4

**Published:** 2023-03-01

**Authors:** Bartosz Bujan, Tobit Fischer, Sarah Dietz-Terjung, Aribert Bauerfeind, Piotr Jedrysiak, Martina Große Sundrup, Janne Hamann, Christoph Schöbel

**Affiliations:** 1 Klinik Lengg AG, Neurorehabilitation Center, Bleulerstrasse 60, 8008 Zurich, Switzerland; 2 Essen University Hospital, Ruhrlandklinik, Tueschener Weg 40, 45239 Essen, Germany; 3 Klinik Lengg AG, Swiss Epilepsy Center, Bleulerstrasse 60, 8008 Zurich, Switzerland; 4 Essen University Hospital, Neurorehabilitation Center, Bleulerstrasse 60, 8008 Zurich, Switzerland

**Keywords:** Medical research, Neurology

## Abstract

Respiratory rate (RR) is an often underestimated and underreported vital sign with tremendous clinical value. As a predictor of cardiopulmonary arrest, chronic obstructive pulmonary disease (COPD) exacerbation or indicator of health state for example in COVID-19 patients, respiratory rate could be especially valuable in remote long-term patient monitoring, which is challenging to implement. Contactless devices for home use aim to overcome these challenges. In this study, the contactless Sleepiz One+ respiration monitor for home use during sleep was validated against the thoracic effort belt. The agreement of instantaneous breathing rate and breathing rate statistics between the Sleepiz One+ device and the thoracic effort belt was initially evaluated during a 20-min sleep window under controlled conditions (no body movement) on a cohort of 19 participants and secondly in a more natural setting (uncontrolled for body movement) during a whole night on a cohort of 139 participants. Excellent agreement was shown for instantaneous breathing rate to be within 3 breaths per minute (Brpm) compared to thoracic effort band with an accuracy of 100% and mean absolute error (MAE) of 0.39 Brpm for the setting controlled for movement, and an accuracy of 99.5% with a MAE of 0.48 Brpm for the whole night measurement, respectively. Excellent agreement was also achieved for the respiratory rate statistics over the whole night with absolute errors of 0.43, 0.39 and 0.67 Brpm for the 10th, 50th and 90th percentiles, respectively. Based on these results we conclude that the Sleepiz One+ can estimate instantaneous respiratory rate and its summary statistics at high accuracy in a clinical setting. Further studies are required to evaluate the performance in the home environment, however, it is expected that the performance is at similar level, as the measurement conditions for the Sleepiz One+ device are better at home than in a clinical setting.

## Introduction

Chronic diseases represent a remarkable health burden. Non-communicable diseases accounted for 74% of deaths globally in 2019, with ischemic heart disease (IHD), stroke, and chronic obstructive pulmonary disease (COPD) being the top 3 leading causes^1^.

Due to their morbidity and mortality, these conditions induce an increasing economic and social burden in a growing and aging population with the demand for innovative approaches to disease management.

Changes in respiratory rate (RR) is a known indicator of serious conditions in lung diseases like cystic fibrosis (CF) or COPD. Recent studies reported that a high RR was the most important predictor of cardiopulmonary arrest in hospitals^2^.

In 2017, COPD was estimated to affect ~ 300 million people worldwide, with a relative increase of 5.9% between 1990 and 2017^3^. It is a preventable disease, characterized by persistent respiratory symptoms. A substantial part of COPD management is the prevention of exacerbations, defined as an acute worsening of symptoms resulting in additional therapy^4^. Within the past decade, it has been—and is still—of great interest to find strategies to diagnose COPD exacerbations earlier and/or prevent them completely, as they impact disease progression negatively. Studies suggest that long-term RR monitoring at home could be a valid tool to predict exacerbations^5–7^, but more investigation is needed to confirm these results and to find an easy-to-use solution for most patients.

With at least 26 million people affected in 2017, heart failure (HF) is another main cause of morbidity in elderly patients^8^. Recently, ambient sensor technology was used in a pilot approach to detect early signs of HF decompensation—among others, an increase in RR, heart rate (HR) and body movement in bed could be detected weeks before decompensation^9^. Even in the prediction of adverse events in IHD itself, RR monitoring is proved to be an integral part of the assessment of cardiac arrest prediction^10^.

Most recently, the COVID-19 pandemic highlighted the importance of RR monitoring, revealing the need for alternative medical solutions, including remote patient monitoring^11^.

Despite the clinical relevance and importance, long-term monitoring of RR faces some difficulties. For instance, just the fact that RR is measured can affect the patients breathing and change results. Moreover, RR is sensitive to various stressors, such as cognitive load, emotional stress, heat, cold, and physical effort^12^.

Non-contact biomotion sensing offers multiple advantages. Sensors can be deployed in a home setting, preferably during nighttime, which can help to eradicate the difficulties mentioned above. Due to their non-intrusive design and no action needed from patients’ side (given the device is placed correctly once), this technology can provide instantaneous RR for several hours in a natural setting. This might be the basis for trend analyses and novel monitoring approaches, combined with other parameters, to constantly advance disease management.

Various contactless approaches have been adopted. Mainly, the technologies can be grouped in four different categories; radiofrequency (RF) based systems and pressure sensor-based systems are already far in development, while audio and visual-based systems are in a more experimental stage. The latter mainly rely on advanced algorithms to extract relevant parameters from either audio signals^13^ or different visual signals^14–18^. Most pressure sensor-based systems use piezoelectric or electromechanical film pressure sensors and consist of a pad that is either placed on or below the mattress of the user during sleep^19–23^.

Finally, various devices and research projects use radiofrequency-based technology.

Doppler radar technology has attracted attention in the medical field as it can detect small amplitude vibration motions caused by breathing excursion and cardiac movement thus can be used for vital sign detection and sleep monitoring^24^. Recent advancements in radar systems have demonstrated their ability to capture physiological signals such as respiration frequency, heart rate and body movement in a contactless fashion^25–28^. Electromagnetic waves travel to the surface of the chest cavity and reflect from it. The antenna receives the reflected waveform containing information on the chest displacement. When the body is at rest, this movement is mainly caused by respiration and heart contractions. With an advanced digital signal processing pipeline, from respiration and heart contraction motions, respiration and heart rate can be estimated in nearly real-time, with very high accuracy. However, to date none of these systems is classified as a medical-grade diagnostic tool, mostly due to the lack of a proper clinical validation.

In this study, we aim to validate a novel contactless breathing monitoring solution based on the Doppler radar technology and advanced signal processing, developed to measure precise movements of the thorax caused by breathing effort and extract RR and breathing patterns for sleep monitoring and diagnosis (Sleepiz One+, Sleepiz AG, Switzerland). The performance of the device in detecting RR was benchmarked in a clinical setting against the thoracic effort belt.

## Methods

### Study design

This paper covers studies validating RR with a contactless sleep monitor (CSM) against the thoracic effort belt (TREB) of the polysomnography (PSG) setup, in two different clinics (Klinik Lengg, Switzerland and Ruhrlandklinik, Germany).

In a monocentric, prospective, open-label non-randomized validation study at Klinik Lengg, the CSM was validated against PSG over 20 min on resting participants and during sleep (Study 1).

Additionally, we analyzed a dataset obtained as part of a larger study at Ruhrlandklinik, where the participants underwent a single overnight recording in the sleep laboratory with a PSG setup and the Sleepiz device (Study 2).

### Participants

In Study 1, patients suspected to suffer from sleep-related disorders or chronic cardiac, respiratory, or neuromuscular disorders were recruited from the neurorehabilitation ward of Klinik Lengg. The subjects included in Study 2 were patients of the Ruhrlandklinik, with a suspected or diagnosed sleep-related breathing disorder.

In both studies, the patients were ≥ 18 years old, and could consent to electrophysiological routine assessment (PSG) in writing. Exclusion criteria were previous enrolment into the current study, being a study investigator or one of their relatives, employees, and other dependent persons, having an implanted electrical device, pregnancy and inability to follow the study procedures, e.g. due to language problems or psychological disorders.

### Sleepiz One+ description

**Figure 1:**
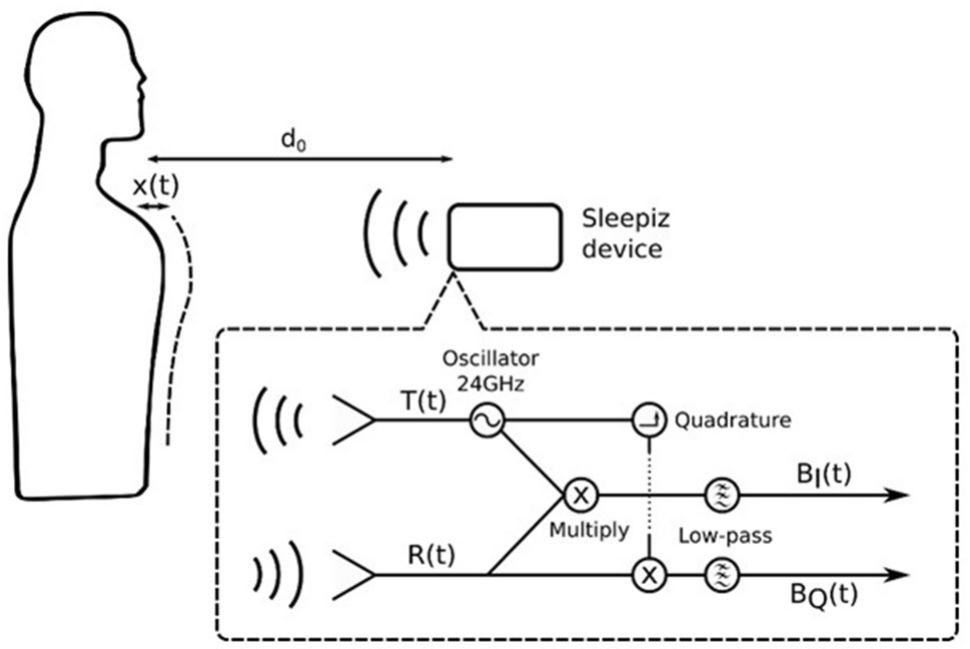
Schematic of radar operation. *T(t)* transmitted electromagnetic signal (f = 24 GHz), *R(t)* reflected signal, *BI(t)* in-phase channel, received signal multiplied by transmitted signal, low-pass filtered, *BQ(t)* quadrature channel, received signal multiplied by 90° offset of transmitted channel, low-pass filtered. Figure provided by Sleepiz AG, Zürich, Switzerland; appeared in unpublished poster related to^47^.

The Sleepiz One+ is a contactless respiration monitor whose operating principle is continuous-wave Doppler radar. The device is placed on the bedside table or a stand beside the user’s bed and points at the user’s thorax from a 40–50 cm distance. From this position, it transmits a continuous electromagnetic signal at a fixed frequency of 24 GHz (output power < 18 dBm) which is reflected at the user’s thorax, while being minimally influenced by clothing or blankets. With a beam aperture of 80° in horizontal- and 34° in vertical plane, the Sleepiz One+’s field of view from the specified position covers the user’s whole thorax-abdomen region. The reflected signal is received and pre-processed using a quadrature receiver^29^, resulting in two output channels BI(t) and BQ(t) which contain information on the movement in the whole thorax-abdomen region as distance changes of the user relative to the measurement device^24,30^ (Fig. 1).

The Sleepiz One+ data is transmitted to a cloud server, where it is analyzed using proprietary software which identifies and extracts rhythmic thorax and abdomen movements related to breathing motions to provide RR estimates, whose validation is the topic of this work.

The Sleepiz One+ is able to operate with two people in the same bed, but records only the respiration rate of the person closest to the device. Furthermore, multiple devices can operate in the same room, as long as their field of views do not intersect. Within this study, all participants were recorded with no other people in the bed, and with one device per room.

### Experimental setup

#### Study 1

**Figure 2:**
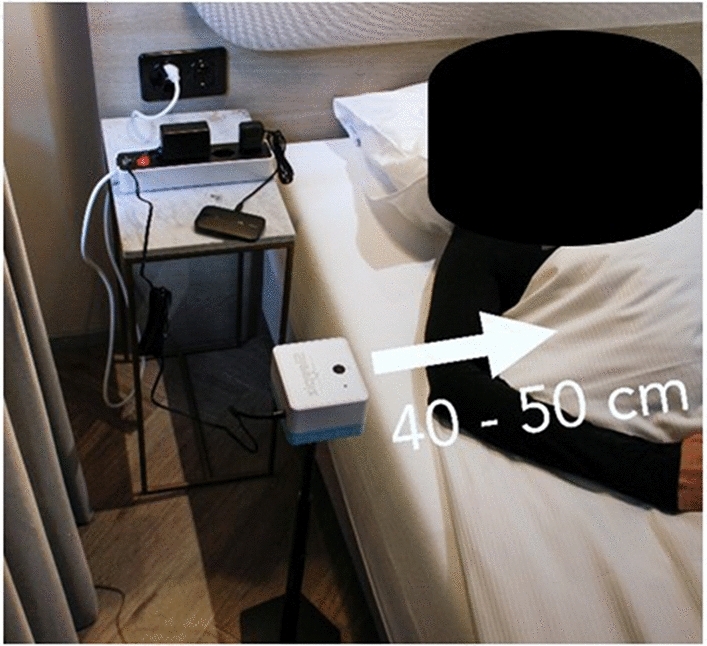
Placement of Sleepiz One+ device.

Each participant was monitored for one night simultaneously with the Sleepiz One+ and a full PSG setup (SOMNOtouch RESP, Somnomedics) including electrocardiogram, electroencephalogram, airflow (nasal cannula), respiratory effort (thorax and abdomen belts) and pulse oximetry. The Sleepiz One+ device was placed beside the bed at a distance of 50 cm pointing at the participant’s thorax (Fig. 2). The person included in the image has provided informed consent to publish the image. All enrolled participants underwent the same procedure.

#### Study 2

Each participant underwent an overnight recording in the sleep laboratory with a full PSG setup (Nox A1, Nox Medical), including a TREB), which was the channel used for the performance assessment. The Sleepiz One+ was placed beside the bed at a distance of 50 cm pointing at the participant’s thorax.

### Statistical analysis

#### Data preprocessing

Data from each device, namely the TREBs of the PSG systems and the Sleepiz device, were exported and converted to a Python dictionary containing its raw data, measurement start time, and sampling rate. Next, devices were time-synchronized with each other. Finally, quality assessment was performed. The signals were visually assessed to identify artifacts, i.e. regions that are not interpretable, due to the patient moving or the sensors being positioned suboptimally, leading to a signal-to-noise ratio insufficient for analysis. These were removed from further analysis. The criteria used to exclude participants for analysis are specified below.

#### Study 1

For each patient, the 20-min analysis window to be included for instantaneous RR performance assessment was selected by identifying a window of 20 min of the recording, where the person was calmly lying in bed, within the first 3 h after the recording setup had been completed. The window was selected based on the following three conditions: (1) the reference device had started recording, (2) the Sleepiz device had started recording, and (3) less than 10 min of total duration where either the patient was moving, as detected by the Sleepiz device, or there were artifacts in RR estimates from the TREB.

The purpose of these conditions was to ensure the correctness of the reference instantaneous RRs and that a sufficient portion of the 20 min could be used for analysis (i.e., it was movement, breath irregularities and artifact-free), also considering that these conditions were most likely to be met in the first 3 h of sleep.

If no analysis window fulfilling all three conditions was identified in the first three hours of the recording, as determined by the Sleepiz device start time, the patient was excluded from the analysis. No sleep stage information was used for the selection of the analysis windows.

Participants were excluded from the RR statistics analysis if either the TREB or the Sleepiz data presented artifacts for 60% or more of the time-points between analysis start and end times. The start time would be the time of device which started to record later of the two and end time would the time of the device ended earlier of the two.

#### Study 2

Exclusion criteria were the same as for Study 1 for RR statistics analysis.

#### Computation of instantaneous respiratory rate

We define instantaneous RR as the number of breaths in a time window of 60 s (epoch), where consecutive time windows have an overlap of 55 s, i.e., they present a 5 s offset.

Instantaneous RR was computed for the data from the TREB through the following procedure:

- The signals were smoothed with a 7th order Butterworth low-pass filter with a cut-off frequency of 0.6 Hz. The filter cut-off was chosen based on the assumption of a maximum RR of 35Brpm.
- The inhalation times were identified by performing a quantile normalization and finding maxima in the resulting signal fulfilling properties related to the peak prominence and minimum distance between peaks. The identified inhalation times were visually reviewed to ensure their correctness.
- The instantaneous RR was derived by taking the inverse of the median time between inhalations.

**Figure 3:**
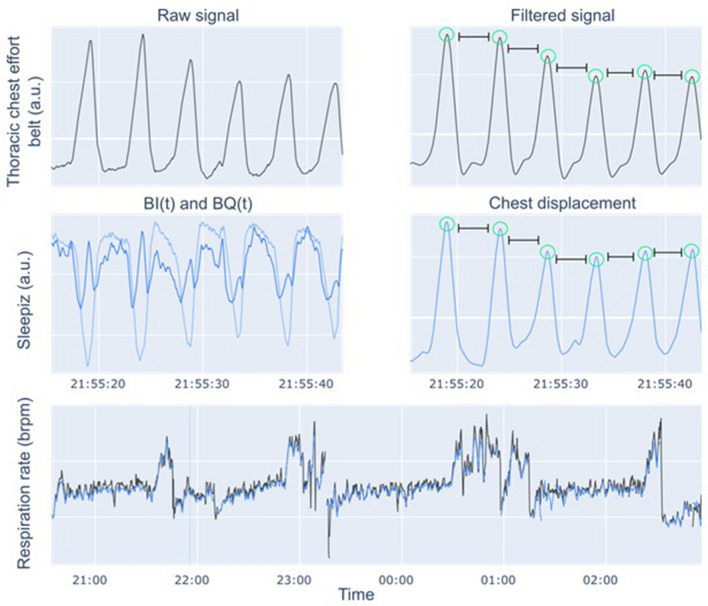
Data processing pipeline. Top row: estimation of instantaneous respiratory rate from thoracic chest effort band (TREB). The raw TREB data, presented in arbitrary units (a.u.) is first smoothed, inhalation times identified, and, from these, instantaneous respiratory rate is computed (see “Computation of instantaneous respiratory rate” for a detailed description). Middle row: estimation of instantaneous respiratory rate from Sleepiz One+ data. The raw signals (BI(t) and BQ(t)), presented in a.u. are used to compute the participant’s chest displacement, from which statistics are extracted and used to compute respiratory rate. Bottom row: the instantaneous respiratory rate for the whole night is used to compute summary statistics, i.e. the 10%, 50%, and 90% percentiles of the respiratory rate distribution.

The computed instantaneous RR from TREB inhalation times was not post-processed through any other means. Exhale times were not used for the RR computation due to challenges in precisely identifying the time of complete exhale; the exhalation curve is frequently flatter than the inhalation curve, and contains multiple peaks (Fig. 3, top row).

RR was computed for the Sleepiz One+ data following a similar procedure. The patient's chest movement was first computed from the raw data from the Sleepiz One+, consisting of channels BI(t) and BQ(t), using an arctangent demodulation algorithm. The chest movement was then filtered and normalized, and the inhalation and exhalation times were identified from the resulting signal. This information was used to compute a probability density over RRs in the range of 5–35 Brpm. In other words, for each RR within the range of 5 to 35 Brpm, the probability of the patient having that particular RR, given the observed inhalation and exhalation times, was computed. The probability density was used to obtain a RR per epoch, as well as to estimate the prediction confidence, i.e., the confidence of the algorithm in the RR prediction made being correct. If the confidence in the prediction is below a threshold, no RR is provided. It is worth noting that times where the patient is tossing and turning are automatically detected by the software and removed from the analysis, as these can lead to errors in the computation of RR.

#### Computation of respiratory rate statistics

RR statistics consisted of the minimum, average, and maximum RR over the recording. To remove the influence of outliers on these metrics, we computed them using the data percentiles, i.e. through the 10%, 50%, and 90% percentiles of instantaneous RR.

#### Performance evaluation metrics

Instantaneous RR performance was assessed through the mean absolute error (MAE), and accuracy: the fraction of time-points where the estimates provided by the reference device and the Sleepiz device were within 3 Brpm.

The agreement between reference and Sleepiz device for RR statistics was evaluated by the absolute error (AE).

#### Sample size

The minimum number of participants required for statistically significant outcomes of instantaneous RR accuracy was calculated using Eq. (1):

1 $${n}_{min}=\frac{\left(Z\alpha \right)2 \cdot {S}^{2}}{d2}$$

where *Z*a determines the confidence level of the estimate (significance level), *S* is the sample standard deviation, and *d* is the minimal effect of interest. The significance level was set to 95%, corresponding to a value of *Z*a to 1.64. The sample standard deviation was set to 5.2%, based on results from a previous pilot study. Finally, the minimal effect of interest was set to 2%. From these, we obtain a sample size of 19 participants. We included more than said number of participants in each center.

### Ethical approval

The authors are accountable for all aspects of the work in ensuring that questions related to the accuracy or integrity of any part of the work are appropriately investigated and resolved. Both included studies were conducted in accordance with the Declaration of Helsinki, the guidelines of Good Clinical Practice (GCP) issued by ICH, as well as the European Regulation on medical devices 2017/745, ISO 14155 and ISO 14971, and the Swiss Law and Swiss regulatory authority’s requirements (Study 1) or the German Law and German regulatory authority’s requirements (Study 2), respectively. Both studies were approved by the local ethics committees (Study 1: BASEC-Nr. 2020-00455, Cantonal Ethics Committee Zurich; Study 2: 19-8961-BO, Ethics committee of the medical faculty of the University of Duisburg-Essen) and written informed consent was taken from all individual participants.

## Results

### Participants

Subjects suspected of suffering from sleep apnea were enrolled in the study based on their availability at the study clinics.

Sleep apnea has a high prevalence in subjects with diagnosed COPD, Asthma, cardiovascular diseases, Diabetes, Parkinson etc. Therefore, these subgroups were represented in the study population.

As per the pre-defined inclusion and exclusion criteria, 24 patients were included in study 1 and, among those, a suitable 20-min time segment meeting the quality criteria outlined above was identified for 19 participants (Table 1). In study 2, 187 subjects were enrolled, and 139 met the quality criteria and were considered for analysis (Table 2).

**Table 1:** demographic information from study 1 participants.

	N	Age [years]	BMI [kg/m^2^]	Parkinson [n]	Stroke [n]	Other [n]
Total	24 (4)	68.2 (14.5)	26.7 (6.1)	5 (1)	15 (2)	4 (1)
Included	19 (3)	67.3 (14.1)	26.6 (4.8)	3 (1)	12 (1)	3 (1)
Excluded	5 (1)	71.8 (17.1)	27.2 (10.3)	2 (0)	3 (1)	1 (0)

The data is segregated into three rows: one with the demographic information over all study 1 participants (Total), one with the demographic information of participants who passed the data quality assessment (Included in analysis), and one with the demographic information of excluded participants (Excluded from analysis). The columns “n” (participant count), “Parkinson”, “Stroke”, “Other” contain the number of participants with diagnosed condition, with the number of females in parenthesis. The columns “Age”, “BMI” (body mass index), specify the mean value, with its standard deviation in parenthesis.

**Table 2:** Demographic information from study 2 participants.

	n	Age [years]	BMI [kg/m^2^]	AHI [events/h]	Asthma [n]	COPD [n]	CVD [n]	Diabetes [n]
Total	187 (65)	59.0 (14.1)	31.8 (6.4)	7.97 (14.0)	12 (8)	18 (10)	115 (38)	32 (9)
Included	139 (51)	57.5 (14.1)	31.0 (6.05)	5.5 (6.33)	11 (7)	12 (8)	85 (31)	23 (8)
Excluded	48 (14)	63.2 (13.2)	34.0 (6.91)	15.1 (24.2)	1 (1)	6 (2)	30 (7)	9 (1)

The data is segregated into three rows: one with the demographic information over all study participants (Total), one with the demographic information of participants who passed the data quality assessment (Included in analysis), and one with the demographic information of excluded participants (Excluded from analysis). The columns “n” (participant count), “Asthma”, “COPD”, “CVD” (cardiovascular diseases: arterial hypertension, coronal arterial disease, heart failure, and heart rhythm disorder), “Diabetes” contain the number of participants with diagnosed condition, with the number of females in parenthesis. The columns “Age”, “BMI” (body mass index), “AHI” (apnea–hypopnea index), specify the mean value, with its standard deviation in parenthesis.

### Instantaneous respiratory rate

The standard for RR monitoring in clinical practice is visual counting of breaths. However, this method has been reported to have a high error^31^. Moreover, counting respiration cycles is not feasible for overnight recordings. Thus, we opted for an automated analysis of the TREB signals. It measures the expansion and contraction of the participant’s chest, the same cue used for assessing respiration visually.

**Figure 4:**
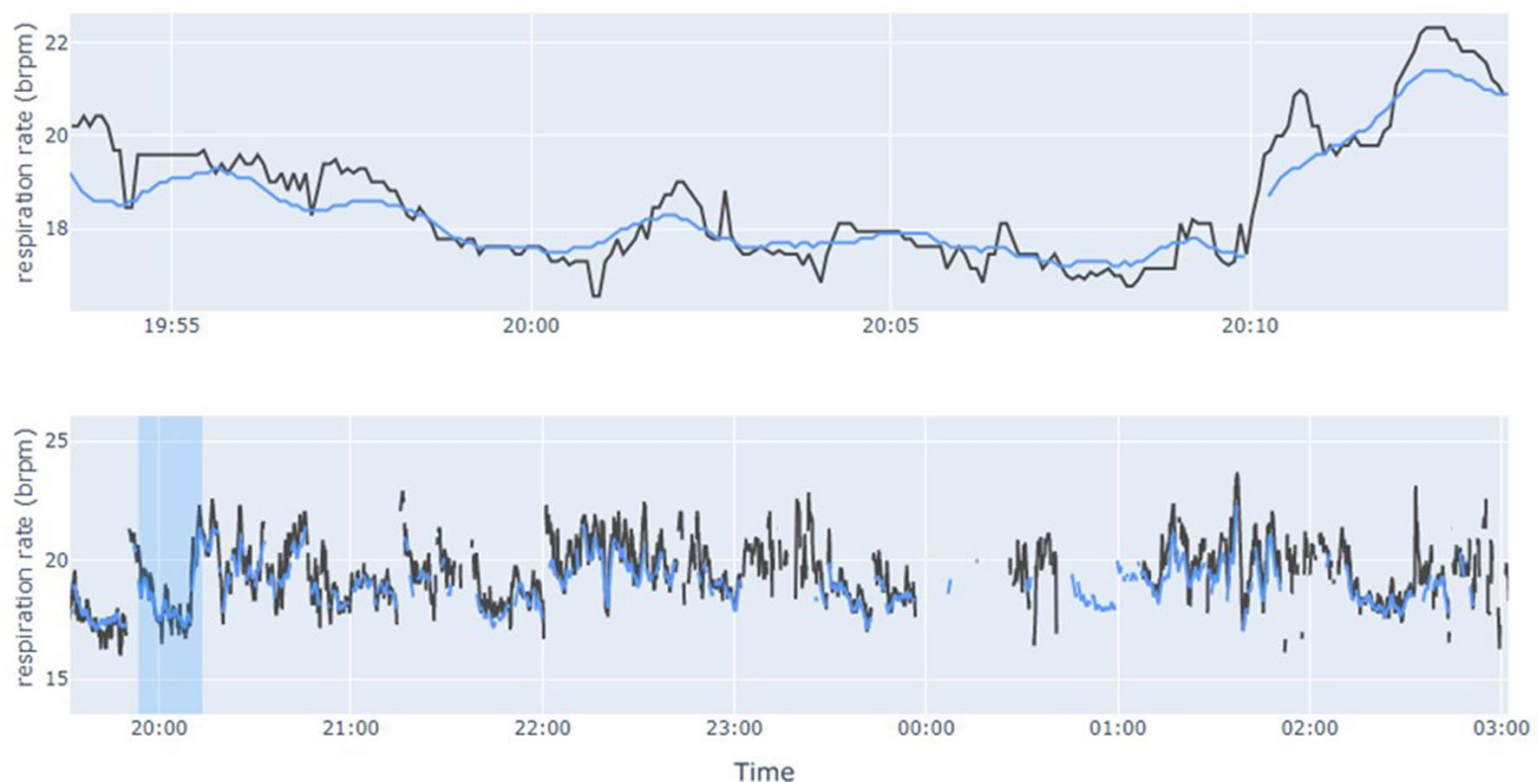
Respiratory rate estimates of a participant from Study 1. Top: instantaneous respiratory rate estimates over the 20-min window used to assess performance. Bottom: respiratory rate over the night, with the 20-min window indicated via blue shading. Estimates from the Sleepiz One+ are presented in blue, and the ones from the thoracic chest effort band in black.

First, we measured instantaneous RR under controlled conditions; participants in study 1 were recorded during a full night in bed with a PSG setup, and a 20-min time segment where they were lying with little or no tossing and turning was selected for the analysis. Figure 4 shows the instantaneous RR estimated during the night for a participant, together with the selected 20-min time window.

**Figure 5:**
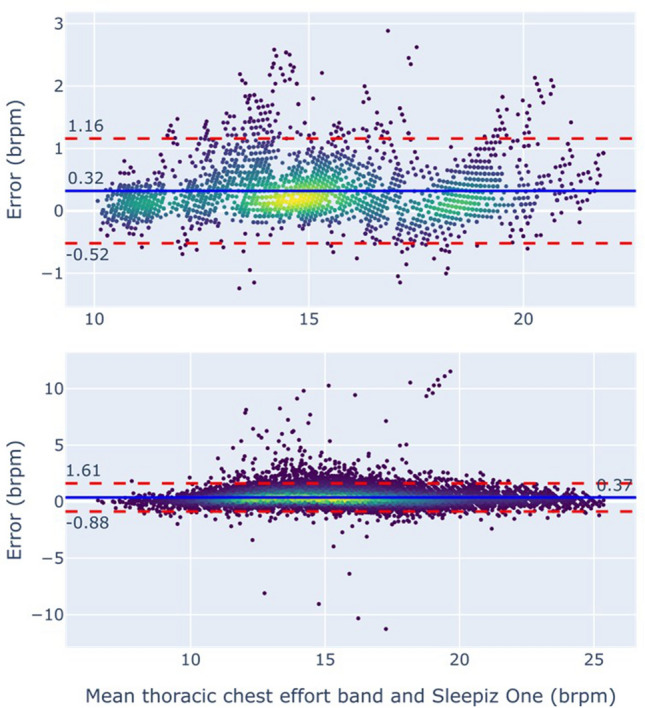
Bland–Altman plot of instantaneous respiratory rate performance. Top: results from Study 1. Bottom: results from Study 2. The solid blue line indicates the bias, while dashed red lines correspond to the 95% limits of agreement.

The data collected in 20-min time segment for 19 participants were assessed. The agreement of instantaneous RR between the TREB and Sleepiz One+ is shown in Fig. 5 and Table 3. The estimates were within 3 Brpm in all timepoints, thus the resulting accuracy of 100%. The MAE had an average value over participants of 0.39 Brpm, and the 95% limits of agreement (LoA) were [− 0.52, 1.16] Brpm (Fig. 5).

**Table 3:** Summary of instantaneous and statistic breathing rate performance.

	Accuracy (%)	MAE (brpm)	LOA (brpm)	AE 10 (brpm)	AE 50 (brpm)	AE 90 (brpm)
Study 1	100% (0%)	0.39 (0.20)	[− 0.52 1.16]	0.41 (0.31)	0.49 (0.26)	0.96 (0.55)
Study 2	99.5% (0.1%)	0.48 (0.14)	[− 0.88 1.61]	0.43 (0.55)	0.39 (0.30)	0.67 (0.52)

The agreement between the instantaneous RR estimates from the TREB and Sleepiz One+ were assessed for the whole night in study 2, where participants spent a night in a sleep lab with full PSG. The results are like those obtained in study 1, with accuracy being 99.5%, average MAE 0.48 Brpm, and the LoA [− 0.88 1.] Brpm (Table 3). Subgroup analysis of instantaneous breathing rate based on the diagnosed conditions in study 2 is shown in Table 4.

**Table 4:** Subgroup analysis of respiratory rate performance of Study 2.

Subgroup	n	Instantaneous respiratory rate		MAE of respiratory rate statistics		
		Accuracy	MAE	10% percentile	50% percentile	90% percentile
COPD	12	99.5% (0.57%)	0.49 (0.21)	0.29 (0.24)	0.29 (0.28)	0.5 (0.31)
Asthma	11	99.3% (1.11%)	0.48 (0.16)	0.4 (0.29)	0.29 (0.21)	0.63 (0.38)
CVD	85	99.5% (0.72%)	0.47 (0.14)	0.45 (0.62)	0.38 (0.32)	0.6 (0.39)
Diabetes	23	99.5% (1.05%)	0.45 (0.14)	0.36 (0.31)	0.37 (0.48)	0.48 (0.34)
Sleep disordered breathing	14	99.5% (0.43%)	0.47 (0.08)	0.45 (0.62)	0.27 (0.21)	0.78 (0.94)
Male	88	99.5% (0.81%)	0.47 (0.14)	0.44 (0.66)	0.42 (0.34)	0.72 (0.59)
Female	51	99.4% (0.83%)	0.48 (0.15)	0.41 (0.26)	0.35 (0.23)	0.57 (0.36)
Total	139	99.5% (0.82%)	0.48 (0.14)	0.43 (0.55)	0.39 (0.3)	0.67 (0.52)

Numbers in parenthesis correspond to standard deviation. Participants can be included in more than one subgroup, as subgroups are defined by the presence of a diagnosed condition, and not by the absence of all other conditions, e.g. a participant with COPD and diabetes is included in the two subgroups.

### Respiratory rate statistics

**Figure 6:**
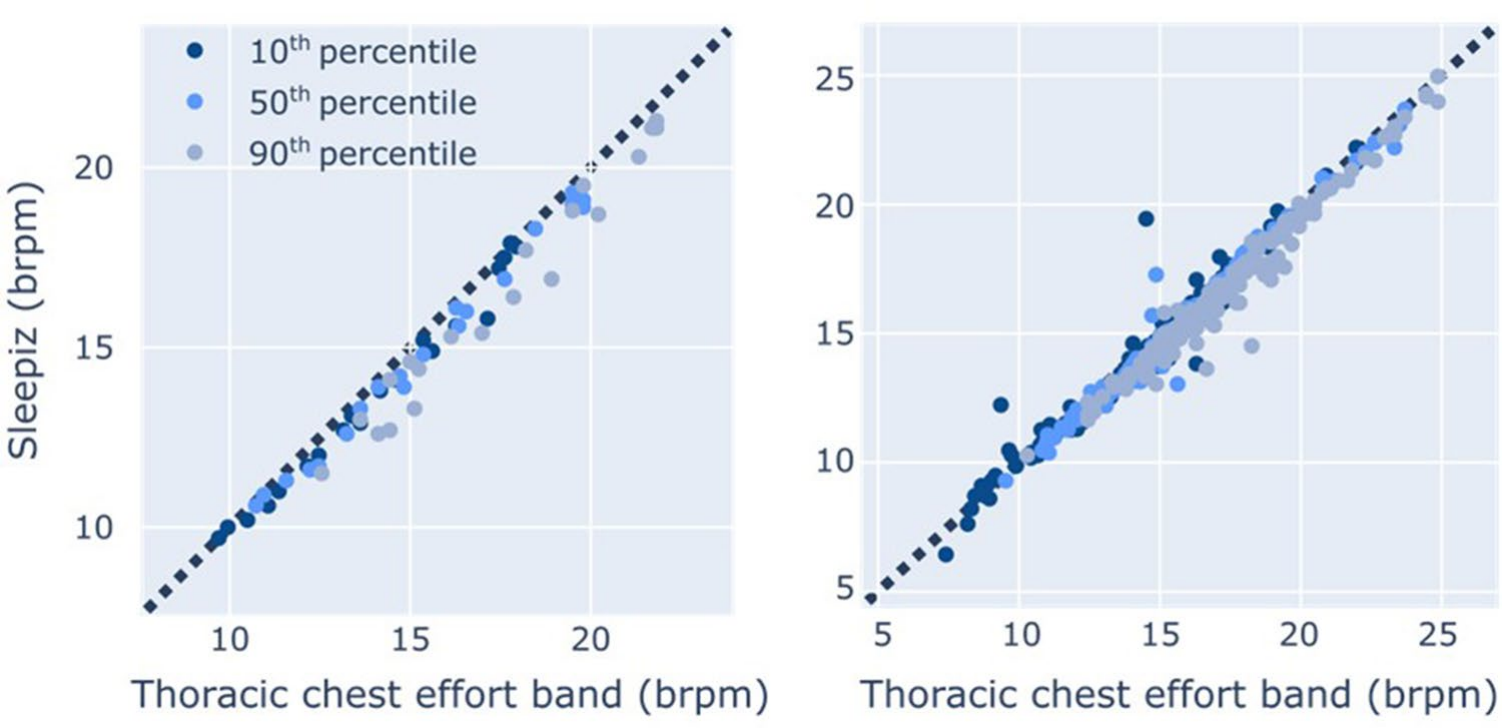
Scatter plot of respiratory rate statistics. Left: Study 1. Right: Study 2.

RR statistics have been shown to be predictive of health status^5–7^. We benchmarked the agreement of RR statistics between TREB and Sleepiz One+, quantified as the 10%, 50%, and 90% percentiles of instantaneous RR over the whole recording (Fig. 6), in study 2 for a total of 139 participants. The results, including a subgroup analysis based on the diagnosed conditions of the subjects, are presented in Table 4.

## Discussion

This report evaluated the accuracy of the Sleepiz One+ in assessing RR; in two separate studies on patients with disturbed breathing.

In study 1, instantaneous RR was assessed in a 20-min time window where participants were lying in bed without significant movement. This setting allowed the assessment of the Sleepiz One+’s performance in absence of abnormal respiration patterns which could confound results. In these conditions, the Sleepiz One+ estimated RR within 3 Brpm for all timepoints and participants included, demonstrating its efficacy in controlled conditions.

The performance of instantaneous RR throughout the night was assessed in Study 2, where patients with suspected or diagnosed sleep-related breathing disorders were monitored throughout the night. In this study, the Sleepiz One+ estimated RR within 3 Brpm in 99.5% of included timepoints.

The two studies evaluated the RR statistics throughout the night, namely the minimum, average, and maximum RR, with the ones obtained from the reference device. The three metrics had an average AE below 1 Brpm in Study 1 and Study 2, indicating that the Sleepiz One+ can accurately measure nightly RR statistics in uncontrolled conditions. This makes the device suitable for its main use scenario, i.e. long-term RR monitoring, that would focus on trend analysis rather than instantaneous RR.

Overall, the two studies yielded similar performance metrics (Table 3) and subgroup analysis showed that there is no notable performance variation between the different diagnosed medical conditions (Table 4). These results provide positive evidence for the accuracy of non-contact respiration monitoring throughout the night in subjects suspected or diagnosed with various sleep-related respiratory disorders, although no further assessment of the comorbidities and their severity was made.

### Comparison to the state of the art

The long-term monitoring of RR in a natural setting enables healthcare professionals (HCPs) to track a patient’s health status over time, which empowers them to take decisions on when the patient should visit the hospital or undergo certain diagnostic procedures. There are other devices in the clinical practice able to measure RR, such as the reference devices used in this study, i.e. TREB and nasal cannula. However, these are not apt for long-term monitoring. There are other certified medical devices that measure RR in a contactless fashion, similar to the Sleepiz One+. These are the RSpot 100 (Kai Medical), SleepMinder (now S+) (Biancamed/Resmed), C100 (Circardia) andXK300 (Xandar Kardian)). The performance of these devices with respect to instantaneous RR estimation is comparable to the one obtained by the Sleepiz One+ (Table 5).

**Table 5:** Performance comparison of instantaneous respiratory rate estimation among radar-based medical devices.

Manufacturer	Device	Confidence bounds (brpm)	References
Sleepiz AG	Sleepiz One+	[− 0.52 1.16]	This report
Circardia	C100	[− 2.3, 1.7]	^44^
Biancamed/Resmed	SleepMinder/S +	[− 1.04, 0.89]	^45^
Kai sensors	Rspot 100	[− 4.5, 1.8]	^46^
Xandar Kardian	XK300	[− 1.7, 1.8]	^41^

The Sleepiz One+ presents several benefits with respect to the solutions available in clinical practice. Firstly, the Sleepiz One+ improves patient comfort during the monitoring of respiratory parameters. Devices such as thoracic respiratory effort bands and nasal cannulas are intrusive and inconvenient. Secondly, the improved comfort, enables long-term respiration monitoring that provides HCPs a more comprehensive picture of the patient’s status and enables them to assess trends and changes. Thirdly, the Sleepiz One+ can be used in a natural setting, as opposed to when contact with the device is required. Outcomes in artificial environments may differ from those obtained in natural ones. Thus, the Sleepiz One+ may provide a more accurate picture of the patient’s status. Finally, HCPs benefit from the convenience and ease of use of the device; minimal involvement is required from their side, and patients can be remotely monitored.

Therefore, we assume that the Sleepiz One+ performance is sufficient for RR monitoring in patients with chronic lung diseases such as COPD, CF or pulmonary fibrosis. Furthermore, the presented results were obtained on patients with disturbed breathing, confirming the device is suitable to be used on patients with a higher likelihood of irregular breathing.

A study by Wilkens et al.^32^ demonstrated that common lung diseases have characteristic breathing patterns that allow early diagnosis. Their change could serve the early detection of possible exacerbations. Cloud-based approaches of telemedicine promise improved patient outcomes, increased physician efficiency and decreased costs^33^. For example, long-term RR monitoring of COPD patients would be helpful as increased RR is a known predictor for exacerbation and hospitalization^34^. Thus, intervention can be provided earlier, and hospitalization times reduced.

In an older study, the accuracy of the non-contact radiofrequency-based biomotion sensor SleepMinder (ResMed Sensor Technologies, Ireland) was investigated in 20 COPD patients^7^. It was observed that SleepMinder estimated RR with good accuracy and therefore is suitable for long-term monitoring providing minimal patient effort. Furthermore, Diraco et al. analysed an ultrawideband radar sensor system for continuous in-home monitoring of 30 older adults^35^. The authors found good overall accuracies between 86 and 96% with a strong dependence on distance and body position. When patients were moving, for example turning around, the accuracy was low due to motion artefacts. Recently, another dual-pulse Doppler system-based approach for the estimation of HR and RR for 20 patients with congestive HF was analysed^36^. The RR estimation median accuracy was 92%, with a median error of 1.2 Brpm. Using a different method, a video-based approach for RR detection relying on automatic region of interest detection for vital sign monitoring was examined^37^. The authors created a benchmark dataset of 148 video sequences obtained on adults under challenging conditions and on neonates, suggesting that video respiratory monitoring provides an encouraging measurement option.

To date, others have proposed radar-technology for vital signs monitoring, showing that the approach yields good results^38–42^. However, these studies are usually done on small patient populations^7,38,41,43^, and how performance varies across patient demographics is not reported.

In this work, we measure the performance of the Sleepiz One+ in a large patient population, reflective of the device’s intended use, and show how the performance is above to that presented in available work^39,42^ and independent of the patients’ pre-existing conditions, such as sleep apnea severity, COPD, CVD, etc.

The results gained with the Sleepiz One+ device are in line with previous findings, indicating that this device is suitable for long time monitoring of RR without impeding patients’ comfort.

#### Limitations

The Sleepiz One+ is intended to be used at the patients’ homes, in addition to the clinic. In the clinic, HCPs are in charge of device setup, while at home the patient is responsible for this task. The effect this has on device performance should be addressed through usability studies where the target users of the device operate it.

Another limitation of conducting the study in a clinical environment and with a PSG as a reference is that participants were subjected to an uncomfortable sleep environment. The Sleepiz One+ , given its contactless nature, allows for natural sleep. We expect device performance to be better in the latter conditions. Due to a reduced frequency of the patients’ movement throughout the night when they sleep in a more comfortable and familiar environment. Whenever patients present significant body movement, the Sleepiz One+ is not able to estimate RR. Thus, at-home measurements have the potential to present a better coverage of the night when RR is available.

To reduce confounding variables, such as apnea events and movement artifacts, a 20-min sleep window within the first 3 h of recording, has been selected to validate the instantaneous RR in Study 1. While this analysis window selection was not related to the subjects’ sleep stage (sleep or awake), it indeed depended on meeting pre-defined inclusion criteria, established to identify 20-min of favourable conditions However, in Study 2, the whole night recordings were analysed to evaluate the instantaneous RR in uncontrolled conditions, which proves the ability of the device to monitor RR during sleep.

A limitation intrinsic to the Sleepiz One+ is its inability to report RR in the presence of significant movement. This could have negative implications for the computation of RR statistics. In this study, RR statistics were computed for TREBs on all epochs where it presented no artifacts, independently of whether the Sleepiz One+ reported a RR estimate at those epochs. In patients with significantly higher variability in RR, a high proportion of body movement could lead to inaccurate RR statistics as estimated by the Sleepiz One+, due to RRs in those epochs not being included in their computation. However, this was not observed in the present study, where the average absolute error of RR statistics was below 1 Brpm (Table 3). Thus, the absence of RR estimates in the presence of tossing and turning does not bias results.

#### Conclusion

The study compared the instantaneous RR and RR statistics between the Sleepiz device and TREB during a 20-min sleep window under controlled conditions and in a more natural setting during a whole night. The results provide evidence for the effectiveness of the Sleepiz device in estimating the instantaneous RR and its summary statistics in the clinical environment. Future studies are planned on evaluating its performance in the home environment and for long-term patient monitoring.

## Data Availability

The data that support the findings of this study are available from the corresponding author upon request.

## Abbreviations

AE: Absolute error
Brpm: Breaths per minute
COPD: Chronic obstructive pulmonary disease
HF: Heart failure
LoA: Limit of agreement
MAE: Mean absolute error
PSG: Polysomnography
RR: Respiratory rate
HR: Heart rate
TREB: Thoracic respiratory effort belt
